# Longitudinal analysis of hsa-miR-3163, hsa-miR-124-3p, hsa-miR-548c-3p, and hsa-miR-27a-3p as prognostic biomarkers in HIV-infected patients

**DOI:** 10.3389/fimmu.2025.1565068

**Published:** 2025-05-06

**Authors:** Ilker Inanç Balkan, Andleeb Shahzadi, Haktan Sönmez, Burhaneddin Oktan, Muhammad Ihtisham Umar, Bilgül Mete, Fehmi Tabak, Günnur Deniz, Umut Can Küçüksezer

**Affiliations:** ^1^ Department of Immunology, Aziz Sancar Institute of Experimental Medicine, İstanbul University, İstanbul, Türkiye; ^2^ Institute of Graduate Studies in Health Sciences, İstanbul University, İstanbul, Türkiye; ^3^ Department of Infectious Diseases and Clinical Microbiology, Cerrahpaşa Faculty of Medicine, Istanbul University-Cerrahpaşa, İstanbul, Türkiye; ^4^ Department of Medical Pharmacology, Cerrahpaşa Faculty of Medicine, Istanbul University-Cerrahpaşa, İstanbul, Türkiye; ^5^ Department of Pharmacy, COMSATS University Islamabad, Lahore, Pakistan

**Keywords:** Human Immunodeficiency Virus (HIV), Antiretroviral Therapy (ART), MicroRNAs (miRNAs), miR-3163 expression, prognostic biomarkers, CD4 T-lymphocyte count, quantitative RT-PCR (qRT-PCR)

## Abstract

**Introduction:**

MicroRNAs (miRNAs), key regulators of cellular pathways, play crucial roles in the pathogenesis of various diseases, including Human Immunodeficiency Virus (HIV). This study aimed to evaluate the expression and diagnostic potential of *in silico*-identified miRNAs (miR-124-3p, miR-27a-3p, miR-548ac-3p, miR-3163) before and during antiretroviral treatment (ART), together with their correlations with immunological markers (CD4 count, CD4/CD45 ratio) and virological parameters (HIV RNA load).

**Methods:**

Blood samples and clinical data of 16 patients were collected at 4 different time points; before the initiation of ART (baseline), 1^st^, 2^nd^ and 6^th^ months following HIV diagnosis. 16 healthy controls were enrolled to this study. RT-qPCR and ELISA techniques were used to analyze miRNA expression levels while immunological markers (CD4 count and ratio) were assessed by flow cytometry.

**Results:**

miR-27a-3p expression was significantly increased at 2^nd^ and 6^th^ months of ART (p<0.001). miR-548ac-3p was upregulated at 6^th^ month compared to healthy individuals and ART-naive subjects (p<0.05). miR-124-3p expression was significantly elevated in ART-naive subjects in comparison with healthy controls (p<0.001). Conversely, miR-3163 was downregulated in ART-naive, 1-month, and 2-month ART groups (p<0.001), but returned to normal levels by 6 months. miR-548ac-3p and miR-3163 showed moderate-to-strong positive correlations with CD4 counts (R=0.46, R=0.67; p<0.001). ROC analysis identified miR-3163 as a promising prognostic marker, with an AUC of 0.8561, (95% CI: 0.756–0.9265).

**Discussion:**

Our findings highlight the potential of miR-3163 as a robust prognostic biomarker for monitoring HIV progression and optimizing ART strategies. Validation in larger cohorts is warranted to confirm its clinical utility.

## Graphical Abstract

The diagram illustrates the role of circulating miRNAs as determinants of treatment response and prognostic indicators in HIV. It highlights the correlation between miRNA levels, CD4 count, and viral load in both naïve and treated patients, with monitoring intervals at 4, 8, and 24 weeks to assess treatment progress.

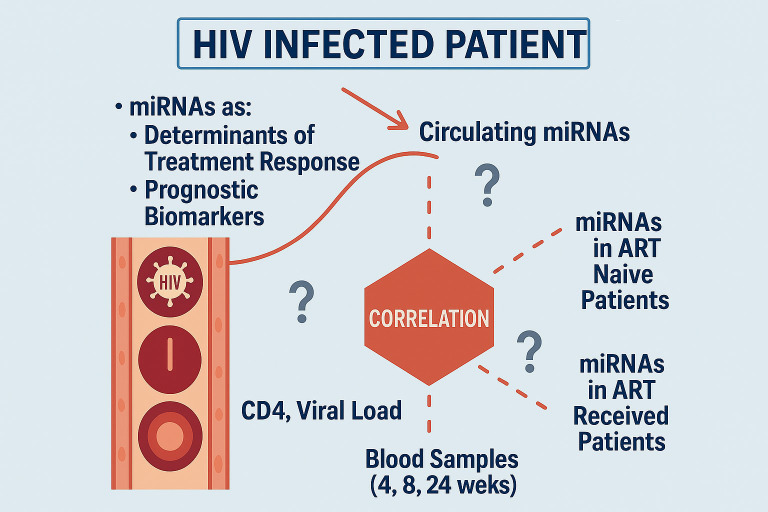

## 1 Introduction

The Human Immunodeficiency Virus (HIV) poses a threat to over 39.9 million people, worldwide (1). Currently, for those living with HIV, the primary and the only evidence-based treatment option is highly active antiretroviral therapy (HAART/ART), which works by inhibiting both the replication and survival of virus at different steps. The progression of HIV infection can vary between individuals, and markers such as viral load, CD4 cell counts, and inflammatory parameters are commonly used to monitor disease progression (2).

MicroRNAs (miRNAs) are small, non-coding RNA molecules consisting of 20-25 nucleotides that play a key role in regulating gene expression (3, 4). Since their discovery, significant progress has been made in cataloging miRNAs, understanding their expression patterns, and identifying their regulatory targets (5). MiRNAs are highly stable in stored patient samples and accurately reflect a patient’s physiological state, making them valuable biomarkers. Their specificity allows them to act as molecular “fingerprints” for diagnosing certain diseases (6).

Research has shown that circulating miRNAs can serve as biomarkers for viral infections such as Ebola and dengue (7–9). Additionally, tissue-specific miRNAs found in plasma show promise as biomarkers for conditions like leukemia, liver damage, and viral hepatitis (10, 11). Cellular miRNAs can directly interact with viruses or their components, influencing viral replication and modifying the body’s response to antiviral treatments (12, 13). A key example is the liver-specific miR-122, which binds to the 5′ untranslated region (UTR) of the hepatitis C virus (HCV) RNA, without altering the stability of viral mRNA. Inhibition of this binding has been shown to increase viral replication (14).

While miRNA research has gained attraction in cancer studies (11, 15), the investigation of miRNA interactions with HIV-1 infection is still relatively new (16). miRNAs play critical roles in HIV pathogenesis, including viral latency, immune activation, and treatment response (17). Only a limited number of studies have explored the diagnostic or therapeutic use of circulating miRNAs in HIV-1 infection (18–21). These studies are often restricted by small sample sizes, limited miRNA analyses, and a lack of independent validation. To date, the miRBase database has cataloged 2,578 human miRNA sequences (Sanger miRBase release 20; http://www.mirbase.org/). During viral infection and replication, host miRNAs and viral RNA interact, regulating protein translation. The differential expression of host and viral miRNAs plays a crucial role in this regulatory process (22, 23). The cellular process of gene silencing, known as RNA interference (RNAi), is mediated by small non-coding RNAs (sncRNAs) and modulates gene expression at the post-transcriptional level (24). This represents a new area beyond traditional treatment strategies. Cellular microRNAs, which regulate protein synthesis, are a key class of sncRNAs that exert effects on multiple biological systems. Given their potential role in viral replication, they could play a significant role in controlling infection and treatment (9, 25).

Recent studies have revealed a connection between miRNAs and the progression of HIV disease (19, 26), It has been found that miR-29 regulates HIV-1 replication and latency (27), miR150 was found to be a potential biomarker of HIV progression and therapy (28) and miR-155 reinforces HIV latency in reservoir cells (29). Similarly, according to the study of Chiang et al. (30) miR-132 increased the HIV-1 replication. However, it remains unclear how miRNA profiles change in HIV patients undergoing antiretroviral therapy (ART) and how these changes correlate with inflammation during HIV progression from an immunological standpoint. Understanding the role miRNAs could lead to the development of new approaches targeting key RNAs or identifying novel protein targets regulated by miRNAs. Gaining insights into these dynamic changes could shed light on HIV-associated chronic inflammation, immune reconstitution, disease prognosis, and potential therapeutic targets.

Since miRNAs in peripheral blood can be easily quantitated using real-time quantitative polymerase chain reactions (RT-qPCR), they hold potential as easily measurable biomarkers for a variety of diseases. In this context, investigation of the correlation between alternations in miRNA levels and shifts in viral load, and immune responses after ART in HIV patients presents a unique opportunity to better understand the chronic inflammation process caused by the disease.

The primary goal of this study was to analyze the levels of peripheral blood miRNAs in newly diagnosed HIV patients before and after ART, assess their correlation with CD4 and CD4/CD45 ratio, evaluate the prognostic potential of miRNA levels, and examine the relationship between miRNA levels and inflammatory complications in HIV patients undergoing long-term ART.

## 2 Materials and methods

### 2.1 Bioinformatics analysis

In our previous research, we conducted a bioinformatics analysis to identify key miRNAs that regulate HIV-related genes. This was done using the multiMiR 1.4 R package, where we screened conserved target regions across 14 prediction databases. We focused on the top 20% of the most frequently predicted targets. We obtained tissue expression data for the predicted miRNAs from the TISSUES 2.0 database, choosing only those with confidence scores higher than 0.5. To study how HIV interacts with host genes, we built a protein-protein interaction (PPI) network using Cytoscape 3.7.2 with data from StringApp. We then used the EnrichR database API for enrichment analysis, applying the Fisher Exact Test and Benjamini-Hochberg corrections to identify important biological processes. Although this bioinformatics analysis is a part of a broader study, the data are not included here (Unpublished).

### 2.2 Study design

This prospective observational study was conducted between 25.04.2022 and 05.12.2024 at Istanbul University, Aziz Sancar Institute of Experimental Medicine, Dept. of Immunology, Istanbul University-Cerrahpaşa, Cerrahpaşa Faculty of Medicine, Department of Infectious Diseases and Clinical Microbiology, and Department of Medical Pharmacology, for the various stages of the research work. The study protocol was approved by the Ethics Committee of Istanbul University-Cerrahpasa (approval number: 25.04.2022-368254), in compliance with the Declaration of Helsinki. The primary aim of the study was to investigate miRNA profiles in HIV-positive patients and validate these findings with a control group.

This study enrolled recently diagnosed, treatment-naive HIV-1 positive patients before and within short term after anti-retroviral treatment (ART) initiation (0- 4^th^ week -8^th^ week - 12^th^ weeks of treatment). Eligable participants were adults aged 18 years or older, who provided written informed consent for blood sampling and storage for future research. A control group of healthy individuals were included for comparison.

Exclusion criteria included pregnancy or breastfeeding, prior initiation of ART at other facilities, and conditions that posed significant risks, such as severe coagulation disorders or impaired venous circulation. Additionally, participants with chronic viral infections else than HIV were also excluded.

In total, 30 HIV-positive patients meeting these criteria were initially enrolled, along with 30 demographically matched healthy controls. However, due to study dropout and participant relocation, complete data with samples from four time points were available for only 16 patients. Consequently, we included 16 healthy controls matched to these patients for the final analysis.

Blood samples for miRNA analysis were collected during patient visits and processed within 30 minutes’ sample integrity (its presented in section 2.4).

### 2.3 Biomarker assessment

The expression analysis of hsa-miR-27a-3p, hsa-miR-124-3p, hsa-miR-3163, and hsa-miR-548c was conducted at Istanbul University-Cerrahpasa, Cerrahpasa Faculty of Medicine, Department of Medical Pharmacology. Blood samples were collected in 10 mL EDTA tubes, and plasma was separated using a double centrifugation method. The expression levels of miRNAs were normalized to RNU6 (U6 snRNA) as the endogenous control. The ΔCt method was used to calculate the relative expression. For relative quantification, the ΔΔCt method was applied using a designated control group as a reference. The fold change in expression levels was determined using the 2^−ΔΔCt method.

All Ct values were recorded, and the mean Ct values from triplicate reactions were used for calculations. Data processing, including ΔCt, ΔΔCt, and fold change calculations, was performed using Microsoft Excel.

### 2.4 Determination of peripheral T cell phenotypes by flow cytometry

The methods in this phase were conducted at Istanbul University, Aziz Sancar Institute of Experimental Medicine, Department of Immunology. Peripheral T cell characterization was performed using flow cytometry. The specific fluorescent labeled monoclonal antibodies were anti-CD45-FITC, anti-CD3-Pacific Blue, and anti-CD4-PE (all antibodies were B&D Biosciences, U.S.). The concentrations of antibodies were optimized prior to the study.

Peripheral blood samples of 100 µL from patients and controls were pipetted into flow cytometry tubes containing the fluorescent labelled monoclonal antibodies and were mixed with pipetting. Then the samples were incubated at room temperature in the dark for 20 minutes. Following incubation, 500 µL of lysing solution (B&D Biosciences, U.S.) was added to remove erythrocytes. Following 15 minutes of incubation in the dark, samples were washed with PBS by centrifugation at 1800 RPM for 5 minutes, then the supernatant was discarded. If necessary, an additional washing step was done in order to obtain cleaner cell pellets. Finally, the cells were resuspended in 500 µL of FACS solution for flow cytometry analysis.

Sample involved acquisition of 10,000 cells within the FSC/SSC lymphocyte gate. White blood cell (WBC) counts from an automated blood counter (Rayto Auto Hematology Analyzer, China) were multiplied by the percentage of CD4-positive cells in the lymphocyte gate of CD4-SSC plot to calculate absolute cell numbers (n/µL). Alternatively, CD4-positive cells in the CD45-SSC plot were quantified as a percentage of lymphocytes, and results were adjusted using WBC counts from the CD45-positive cells.

The study was converted with an ACEA Novocyte flow cytometer running Novoexpress software (Agilent, USA).

### 2.5 Statistical analysis

miRNA expression data were transformed into fold change values for statistical analysis. The normality of quantitative variables was assessed using the Shapiro-Wilk test, and descriptive statistics were reported as mean ± standard deviation.

For statistical comparisons, different tests were applied based on data distribution. In paired samples, the Wilcoxon test was used for non-normally distributed data. When comparing more than two groups, One-way ANOVA was used for normally distributed data, while the Kruskal-Wallis test was applied for non-normally distributed data.

To analyze the combined effects of miRNA expression and treatment time, ANCOVA was performed to examine their influence on CD4 count, while adjusting for potential confounders. In this analysis, Group refers to different treatment time points or patient subgroups, allowing an evaluation of how CD4 count changes over time in relation to miRNA expression levels.

Statistical analyses were conducted using Python (Pandas v2.0.3, Statsmodels v0.14.0), R (version 4.3.2), and Jamovi 2.3.28 within a Jupyter interactive environment. A p-value < 0.05 was considered as statistically significant.

## 3 Results

### 3.1 Demographic data

The patient characteristics are summarized in **Table 1**. The study sample was predominantly male, with 15 male participants (94%) and only 1 female participant (6%). Regarding marital status, 75% of the participants were single, while 25% were married. The participants had neither comorbidities nor opportunistic infections nor malignancies.

**Table 1:** Patient characteristics.

Variable	Frequency	Relative Frequency (%)
Gender		
Female	1	6%
Male	15	94%
Marital Status		
Married	4	25%
Single	12	75%
Route of HIV Infection		
Homosexual	2	13%
Infected Partner	1	6%
Substance Use	3	19%
Unprotected Sex	10	63%
Education		
High School	9	56%
Primary School	6	38%
University Graduate	1	6%
Occupation		
Businessman	1	6%
Student	1	6%
Unemployed	7	44%
Laborer	7	44%
Sexual Orientation		
Heterosexual	3	19%
Homosexual	3	19%
No Information Provided	10	63%
Alcohol Use		
No	7	44%
Yes	9	56%
Smoking Status		
No	5	31%
Yes	11	69%

Regarding the routes of HIV transmission, it was found that 63% of the participants acquired HIV through unprotected sexual intercourse, making it the most common mode of transmission. This was followed by substance use (19%), homosexual relationships (13%), and transmission from an infected partner (6%).

In terms of education levels, the majority of participants (56%) had graduated from high school, 38% had completed primary school, and 6% had received university education.

Looking at their occupations, 44% of the participants were unemployed. A smaller portion of the participants included businessmen (6%) and students (6%). Regarding smoking habits, 69% reported being smokers, while 31% indicated they did not smoke. As for alcohol consumption, it was found that 56% of participants consumed alcohol.

### 3.2 Expression of circulating particular miRNAs in patients with HIV before and after ART

**Figure 1:**
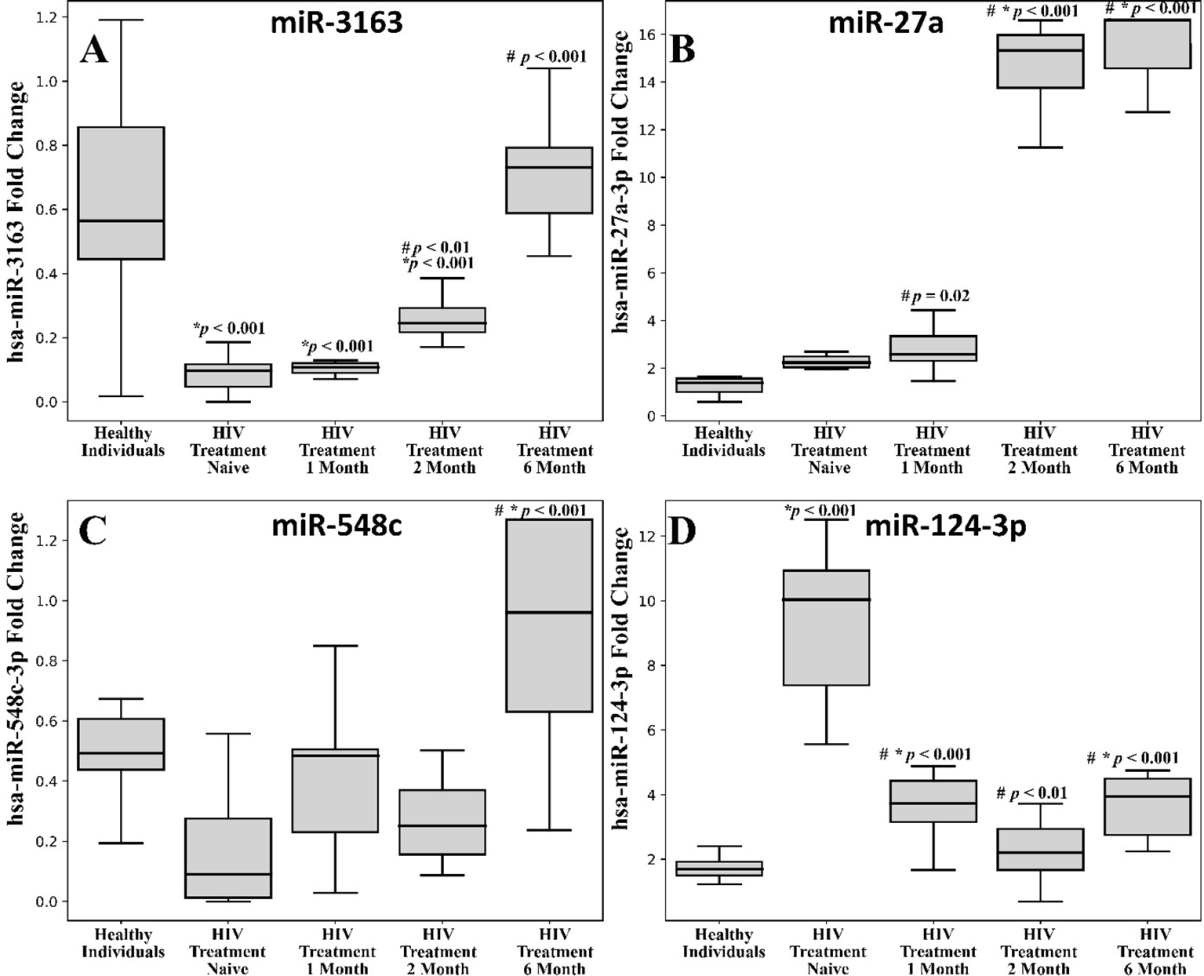
Comparison between the different expression levels of the selected miRNAs among the HIV infected patients and healthy controls. **(A)** miR-316; **(B)** miR-27a; **(C)** miR-548c; **(D)** miR-124-3p. The blood samples were withdrawn from the subjects at different time intervals i.e., day zero, 1 month, 2 months and 6 months of antiviral treatment. Each box plot represents the interquartile range (IQR) of the fold change in the expression of miRNA levels. The middle line in each box shows the 50th percentile (median) whereas the lower and upper boundaries represent the 25th and 75th percentiles respectively. The error bars represent 1.5 times IQR below and above the 25th and 75th percentile respectively to identify outliers. The median values of miRNAs in the different groups of the subjects were statistically compared using one-way analysis of variance (ANOVA) followed by Tukey’s *post hoc* analysis and the differences were considered to be significant at p < 0.05. #comparison with HIV Treatment-Naïve, *Healthy individuals.

We compared the relative expressions (fold changes) of specific miRNAs across different groups of HIV-positive patients, including treatment-naive patients and those at one, two, and six months post-ART, with healthy controls. Significant differences were observed in the expression levels of miR-27a-3p, miR-3163, miR-548c, and miR-124-3p (**Figure 1**).

#### 3.2.1 hsa-miR-3163

Treatment-naive patients (regardless of viral load) showed a significant downregulation of hsa-miR-3163 compared to the control group (*p < 0.001). One-month and two-month post-ART groups exhibited a notable decrease in hsa-miR-3163 expression compared to the healthy individual group (**Figure 1A**, *p < 0.001). Post treatment 2 month and 6 month showed significant upregulation when compared with HIV treatment naïve group (**Figure 1A**, #p < 0.001).

#### 3.2.2 hsa-miR-27a-3p

One-month post- treatment showed upregulation of expression in comparison to HIV treatment naïve group (p = 0.02). While, two-month and six-month post-ART groups exhibited a significant increase in hsa-miR-27a-3p expression compared to the healthy control and treatment naive group (**Figure 1B**, *,#p < 0.001).

#### 3.2.3 hsa-miR-548c

Six-month post-ART group exhibited a significant increase in hsa-miR-548c expression compared to the healthy individual and HIV treatment naive group (**Figure 1C**, *#p < 0.001).

#### 3.2.4 hsa-miR-124-3p

We found a significant downregulation in the hsa-miR-124-3p expression of month 1, 2 and 6 post treatment when compared with HIV treatment naïve group (**Figure 1D**, #p < 0.001). Treatment-naive HIV-positive patients showed a significant upregulation of hsa-miR-124-3p compared to the control group (**Figure 1D**, *p < 0.001). The one-month post-ART group also demonstrated a marked increase in hsa-miR-124-3p expression relative to the healthy individual and HIV treatment naïve group (**Figure 1D**, #*p < 0.001). While the six-month post-ART group exhibited increased expression of hsa-miR-124-3p than that of healthy individuals (**Figure 1D**, *p < 0.001).

#### 3.2.5 miRNA expression across age groups and treatment durations

**Figure 2:**
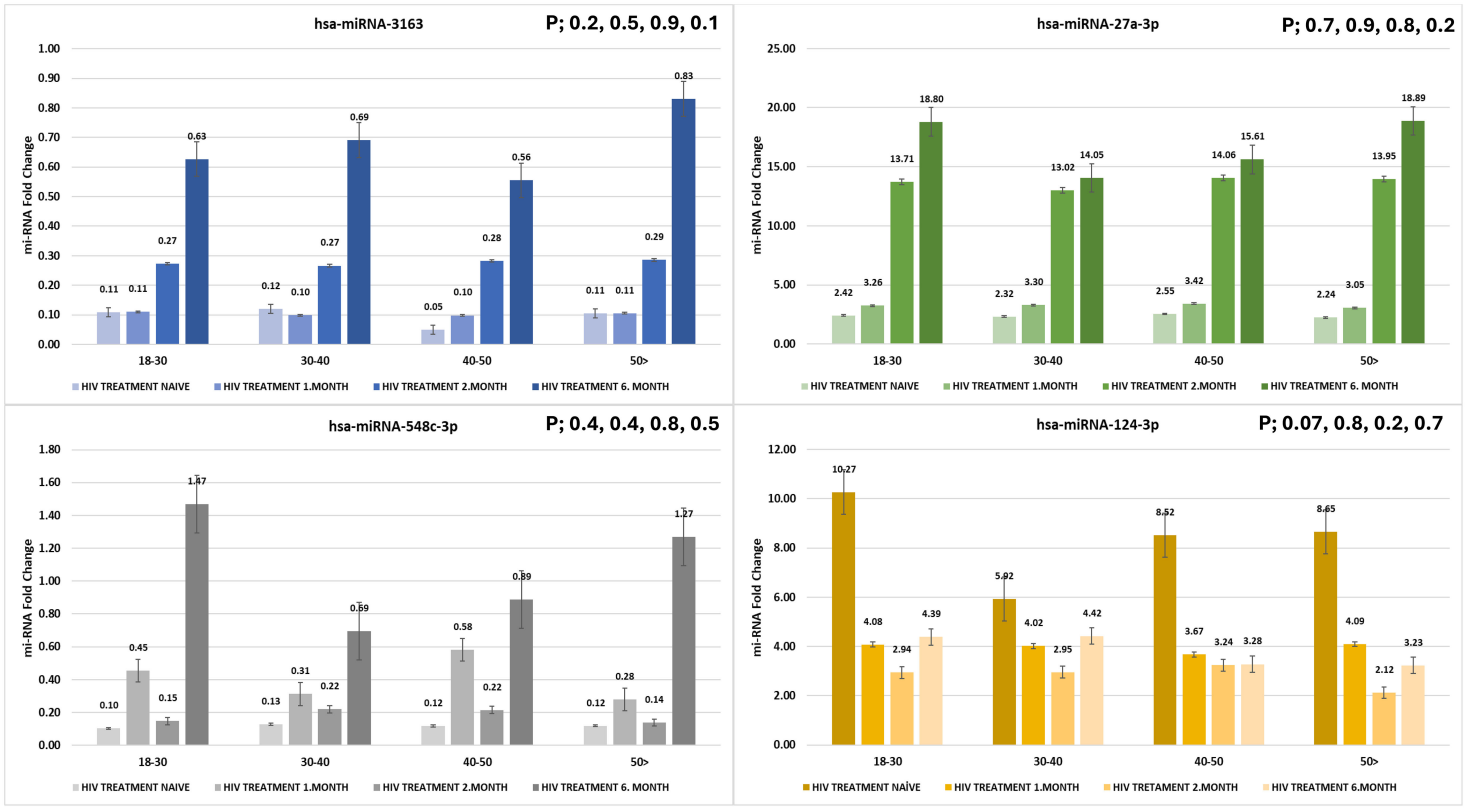
Expression levels of hsa-miR-3163, hsa-miR-27a-3p, hsa-miR-548c-3p, and hsa-miR-124-3p across different age groups and treatment durations. miRNA fold change was evaluated in HIV treatment-naïve individuals and at 1, 3, and 6 months post-treatment (p > 0.05).

The expression levels of hsa-miR-3163, hsa-miR-27a-3p, hsa-miR-548c-3p, and hsa-miR-124-3p were measured across different age groups and treatment durations (HIV treatment-naïve individuals, 1, 2, and 6 months post-treatment). Although the differences were not statistically significant (p > 0.05), noticeable expression patterns were observed across the groups (**Figure 2**)

### 3.3 CD4 cell count recovery following HIV treatment

**Figure 3:**
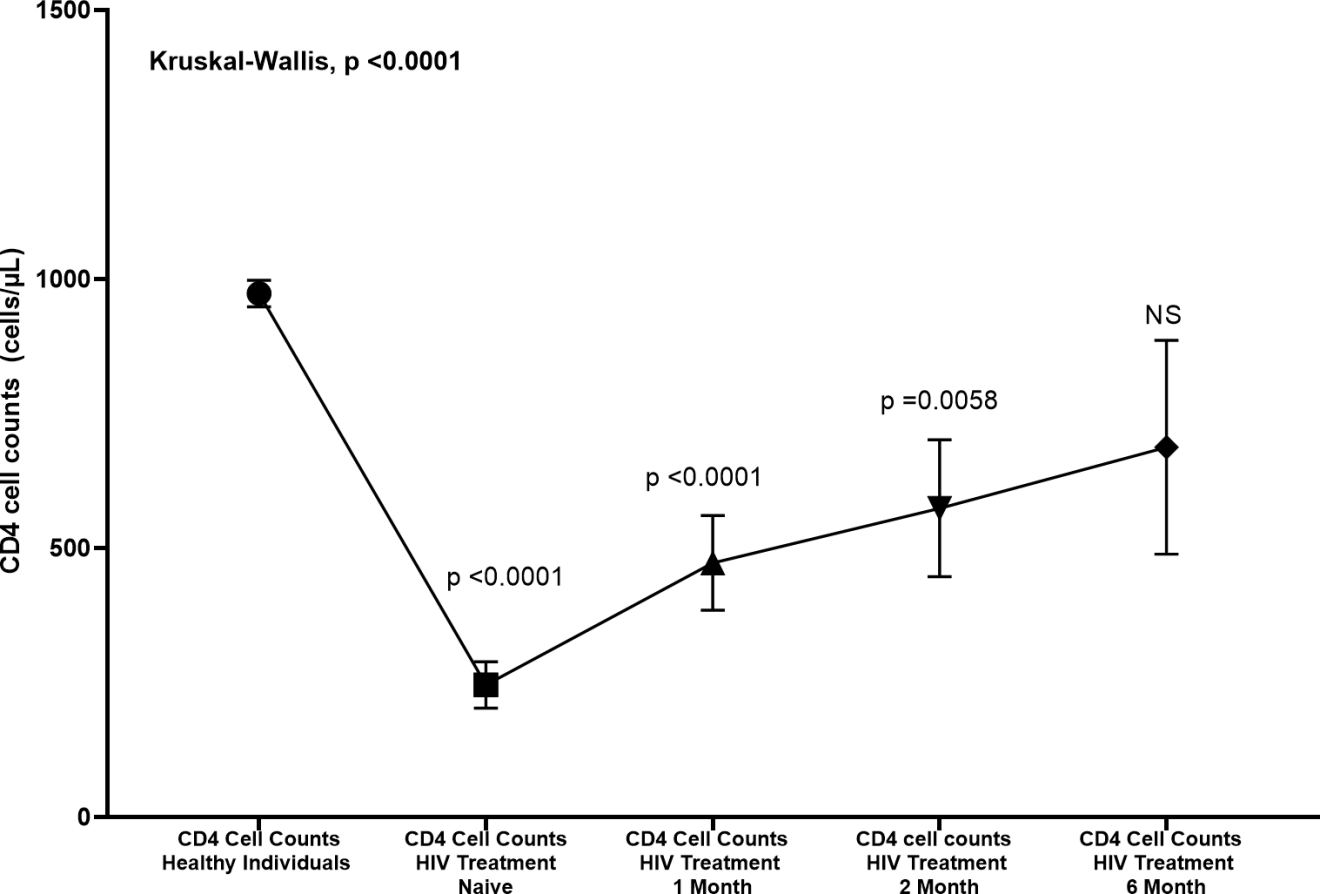
Changes in CD4 cell counts (cells/µL) in HIV patients treated at various time points compared to healthy individuals. CD4 counts were measured at baseline (Day Zero), 1 month, 2 months, and 6 months post-treatment. The Kruskal-Wallis test revealed significant differences between healthy individuals and HIV treatment naive, 1 month, and 2 months (p < 0.0001, p < 0.0001, and p = 0.0058, respectively). No significant difference was observed at 6 months (NS). Error bars represent standard deviation.

In our study we found that CD4 cell counts (**Figure 3**) were significantly reduced in HIV treatment-naïve individuals compared to healthy controls (p < 0.0001). Following the initiation of treatment, CD4 counts showed a significant increase after one month (p < 0.0001 compared to treatment-naïve individuals), demonstrating early immune recovery. This upward trend continued at two months, with a further significant increase (p = 0.0058). However, by six months, although CD4 counts continued to rise, the change was no longer statistically significant (NS).

### 3.4 Correlation analysis

**Figure 4:**
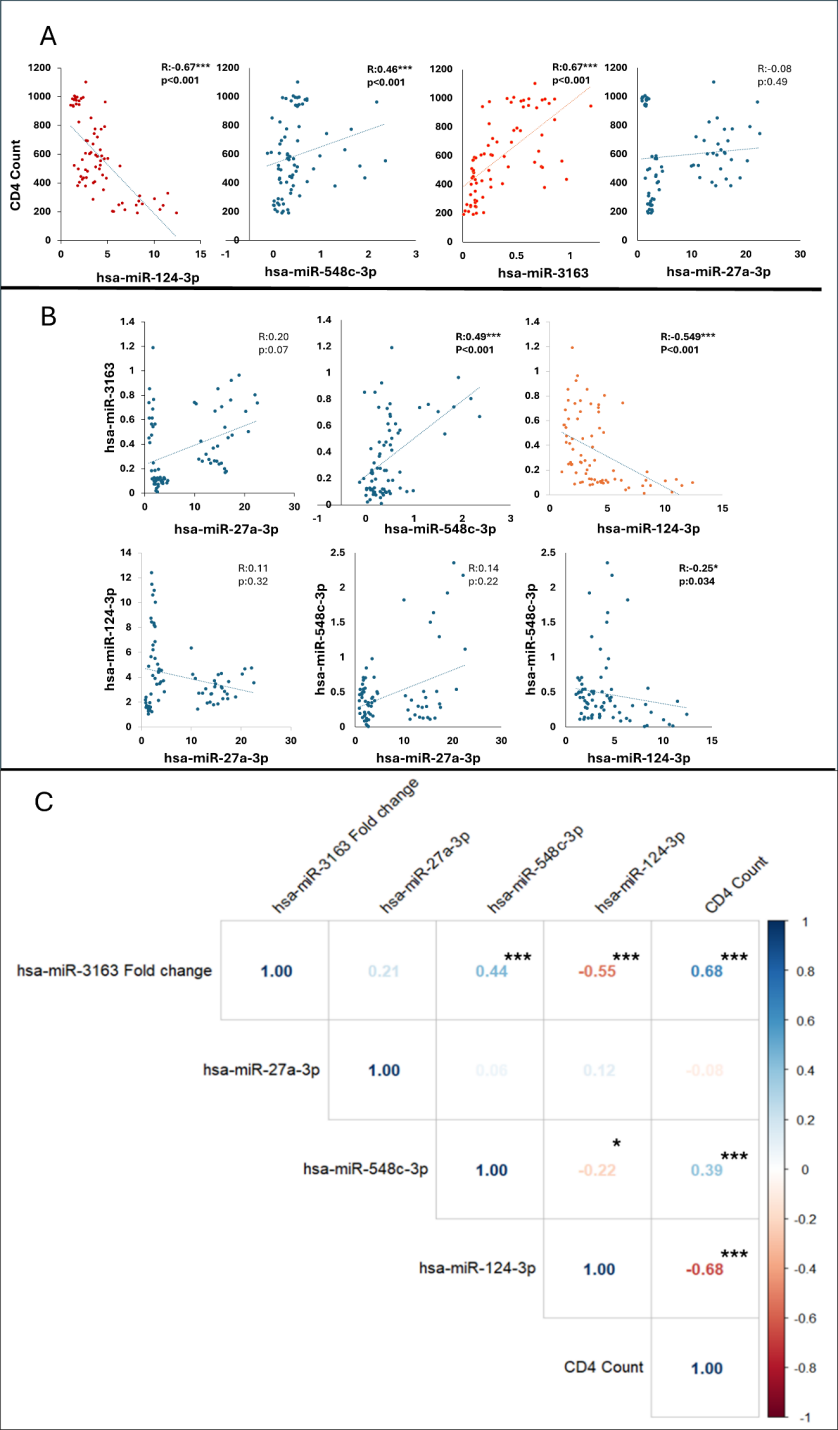
**(A)** The graph shows the correlation between CD4 count and microRNA levels with a trendline (regression line), **(B)** The graph shows the pairwise correlations between microRNAs with a trendline (regression line). *p <.05, ***p <.001. Blue dots indicate that the correlation coefficient r is less than 0.5, and red dots indicate that the correlation coefficient R is greater than 0.5. **(C)**. Correlation heatmap illustrating the relationships between hsa-miR-3163, hsa-miR-27a-3p, hsa-miR-548c-3p, hsa-miR-124-3p, and CD4 count. The Pearson correlation coefficients are represented, with color intensity indicating the strength and direction of correlations (blue for positive and red for negative correlations).

Spearman correlation analysis was employed to assess the relationship between CD4 count and the expression levels of four different miRNAs (**Figure 4**). miRNA-124-3p, miRNA-548c-3p, miRNA-3163, and miRNA-27a-3p. This non-parametric method was chosen due to the non-normal distribution of the data and is suitable for evaluating the strength and direction of monotonic relationships between two variables. A moderate-to-strong negative correlation was observed between miRNA-124-3p expression and CD4 count (R = -0.67, p < 0.001). Moderate and moderate-to-strong positive correlations were identified between CD4 count and the expression of miRNA-548c-3p (R = 0.46, p < 0.001) and miRNA-3163 (R = 0.67, p < 0.001), respectively. Our results depicted no statistically significant correlation between miRNA-27a-3p expression and CD4 count (R = -0.08, p = 0.49). A moderate negative correlation was observed between the expressions of miRNA-124-3p and miRNA-3163 (R= - 0.549, p < 0.001).

**Figure 5:**
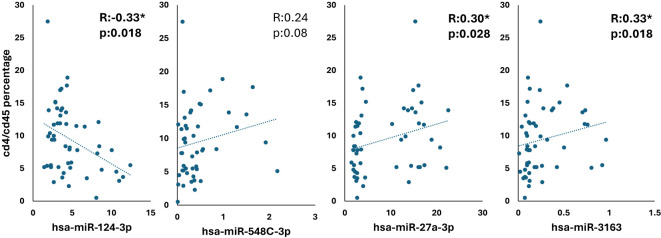
Correlation analysis of CD4/CD45 ratio with miRNA expression in HIV-positive patients *p <.05.

These correlation graphs as shown in **Figure 5** examine the relationship between the CD4/CD45 ratio and four different miRNAs (hsa-miR-124-3p, hsa-miR-548c-3p, hsa-miR-27a-3p, and hsa-miR-3163). A weak negative correlation was observed between hsa-miR-124-3p and the CD4/CD45 ratio (R= - 0.33, p = 0.018), while weak positive correlations were found with the other three microRNAs (hsa-miR-548c-3p: R = 0.24, p = 0.08; hsa-miR-27a-3p: R = 0.30, p = 0.028; hsa-miR-3163: R = 0.33, p = 0.018).

To identify potential interactions between microRNAs and treatment duration in influencing CD4 count (**Table 2**), we employed advanced one-way ANCOVA. Our analysis revealed that two miRNAs (hsa-miR-3163 and hsa-miR-124-3p), which are strongly correlated with CD4 count, significantly affect CD4 count while accounting for the duration of treatment.

**Table 2:** ANCOVA analysis of the effects of group and hsa-miR-3163 on CD4 count: main effects and interaction.

	Sum of Squares	df	Mean Square	F	p	η²
Overall model	1.58e+6	7	225129	13.706	<.001	
hsa-miR-3163	804970	1	804970	49.008	<.001	0.343
Group(treatment time)	750426	3	250142	15.229	<.001	0.320
hsa-miR-3163 Group	20504	3	6835	0.416	0.742	0.009
Residuals	771991	47	16425			

The effects of hsa-miR-3163 and treatment time (Group) on CD4 count were assessed using ANCOVA (**Table 2**). We found that overall model (independent and dependent variables) was statistically significant, F (7, 47) = 13.706, p < 0.001. The main effect (influence on dependent variable i.e. CD4 count)) of hsa-miR-3163 was highly significant (F (1, 47) = 49.008, p < 0.001, η² = 0.343. Likewise, we found the main effect of group (treatment time) was significant (F (3, 47) = 15.229, p < 0.001, η² = 0.320), indicating that CD4 count varied significantly across different treatment durations.

However, the interaction between hsa-miR-3163 and group was not significant (F (3, 47) = 0.416, p = 0.742, η² = 0.009), suggesting that the relationship between hsa-miR-3163 levels and CD4 count remains consistent across treatment time groups. In other words, the influence of “hsa-miR-3163” on CD4 count is consistent regardless of the group.

The Group variable (treatment time) has a statistically significant effect on CD4 count (F = 50.014, p < 0.001; **Table 3**). The hsa-miR-124-3p variable does not have a statistically significant effect on CD4 count (F = 1.955, p = 0.169) indicating that its levels alone do not influence CD4 count. Additionally, the interaction between Group and hsa-miR-124-3p was not statistically significant (F = 1.10, p = 0.359) suggesting that the effect of hsa-miR-124-3p on CD4 count is consistent across the all groups. Based on these results, treatment time (Group) plays a crucial role in determining CD4 levels, while hsa-miR-124-3p does not appear to be a major independent predictor.

**Table 3:** ANCOVA analysis of the effects of group and hsa-miR-124-3p on CD4 count: main effects and interaction.

	Sum of Squares	df	Mean Square	F	p	η²
Overall model	1.60e+6	7	228216	14.29	<.001	
Group(treatment time)	1.52e+6	3	505124	31.64	<.001	0.645
hsa-miR-124-3p	29530	1	29530	1.85	0.180	0.013
Group hsa-miR-124-3p	52612	3	17537	1.10	0.359	0.022
Residuals	750376	47	15965			

### 3.5 The prognostic potential of plasma miRNAs

**Figure 6:**
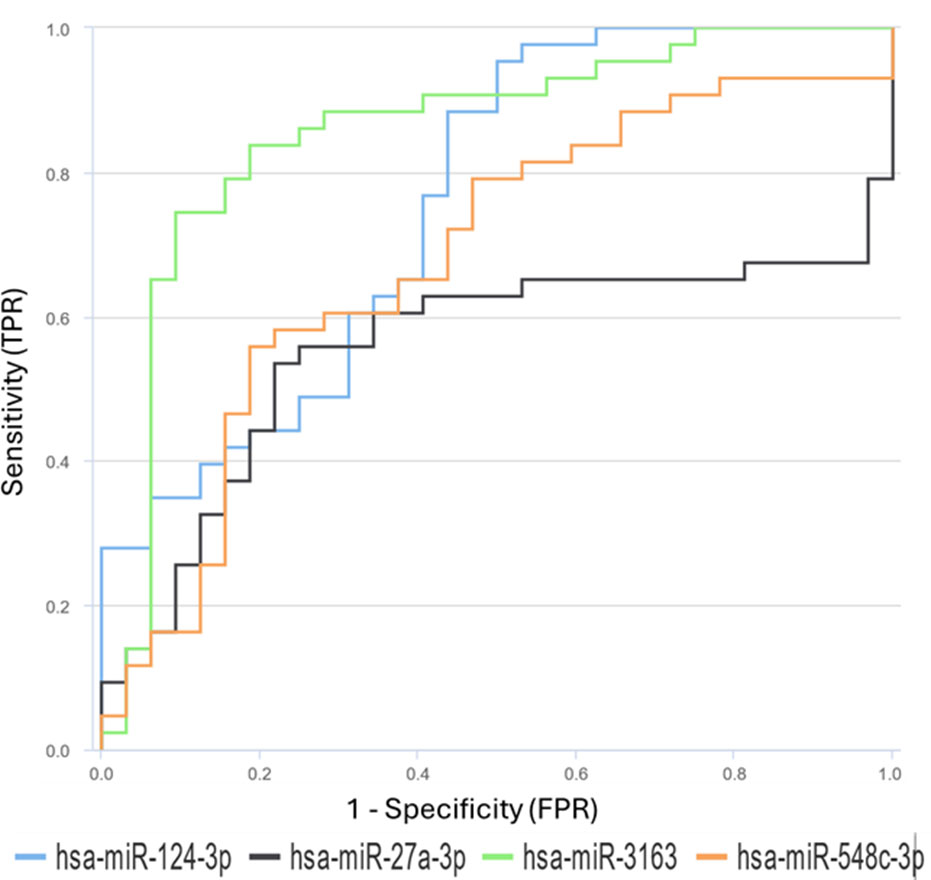
Receiver Operating Characteristic (ROC) curves for differentially expressed miRNAs between HIV-infected individuals and healthy control groups. The ROC curves for miR-27a, miR-3163, miR-548c, and miR-124-3p demonstrate their prognostic effectiveness.

This study aimed to evaluate serum miRNAs as potential markers for monitorization of HIV prognosis (**Table 4**, **Figure 6**). Patients were divided into two groups according to their CD4 counts (cells/μL): CD4 > 500 (“good prognosis”) and CD4 < 500 (“poor prognosis”). The prognostic accuracy of four different miRNAs (hsa-miR-3163, hsa-miR-124-3p, hsa-miR-548c-3p, and hsa-miR-27a-3p) was evaluated using ROC analysis. To determine the optimal cut-off values for each miRNA, the Youden index method was employed.

**Table 4:** miRNAs as potetial markers for monitorization of HIV prognosis and receiver operating characteristic (AUC-ROC) analysis result.

miRNA	AUC level	Prognostic value	Cut-off value (Youden İndex)
hsa-miR-3163	0.85	GOOD	0.263
hsa-miR-124-3p	0.75	MODERATE	0.453
hsa-miR-548c-3p	0.67	POOR	0.426
hsa-miR-27a-3p	0.55	POOR	9.97

hsa-miR-3163 showed the highest prognostic accuracy with an AUC value of 0.8561(95% CI: 0.756 - 0.9265) and an optimal cut-off value of 0.263. This result suggests that hsa-miR-3163 may be a potential marker for monitoring HIV infection prognosis. hsa-miR-124-3p showed moderate prognostic accuracy with an AUC value of 0.7573(95% CI: 0.6445 - 0.8489) and a cut-off value of 0.453. hsa-miR-548c-3p and hsa-miR-27a-3p showed lower prognostic accuracy with AUC values of 0.6795 and 0.5545, and cut-off values of 0.426 and 9.97, respectively.

## 4 Discussion

In the present study, significant alterations in circulating miRNA profiles were observed among HIV-positive patients before and during ART, highlighting their potential role as biomarkers of HIV disease progression and treatment response. miR-27a-3p and miR-548c showed notable increases post-treatment, particularly evident at six months. The expression of miR-124-3p was complex; it was elevated significantly in treatment-naïve patients, peaked at one-month post-ART, and then declined at subsequent time points, indicating its potential dynamic role in modulating immune activation and subsequent normalization during treatment.

To the best of our knowledge, this is the first study to evaluate the relationship between the miR-3163 and key clinical parameters (CD4 and CD4/CD45 ratio) during the follow-up of the same patient group, both before and after antiretroviral therapy. Our findings demonstrate significant changes in circulating miR-3163 expression in HIV-positive patients, with a notable downregulation in treatment-naïve individuals that recovers and becomes significantly upregulated following ART initiation. Although, the function of miR-3163 has not been extensively explored in HIV infection, previous studies in breast (31, 32), cervical (33), colorectal (34, 35) and nasopharyngeal carcinoma (36) have shown its critical role in regulating inflammation and cell survival pathways. In another study, miR-3163 has been identified as a tumor suppressor in hepatocellular carcinoma (HCC) through inhibition of ADAM metalloprotease domain-17 (ADAM17), leading to decreased activation of the Notch signaling pathway (37). Interestingly, recent evidence suggests that despite effective viral suppression by ART, persistent immune activation and low CD4 T-cell counts are associated with elevated plasma extracellular vesicles (pEV), enriched with inflammatory mediators such as ADAM17 (38). Given that it is speculated that miR-3163 expression observed in our study may reduce ADAM17-mediated inflammation.

In another study, it is found that the long non-coding RNA Meg3 enhances miR-3163-mediated suppression of Skp2 protein translation, inhibiting cancer cell growth in NSCLC (39). Skp2, is an ubiquitin ligase essential for HIV Tat-mediated transcriptional elongation (40). Thus, our findings suggest that upregulation of miR-3163 may suppress Skp2, thereby reducing Tat-mediated HIV transcriptional elongation and ultimately inhibiting HIV replication. Taken together, our findings position miR-3163 as a promising candidate biomarker for immune restoration and therapeutic targeting to improve clinical outcomes in HIV-infected individuals on ART.

The observed correlations in our study, between miRNAs and CD4 counts underscore the biological relevance of these molecules in modulation of immune functions. Notably, hsa-miR-3163 showed a robust correlation with CD4 count, positioning it as a strong candidate for further clinical validation. A key strength of our study is the longitudinal follow-up of treatment-naive patients, initially identified as HIV-positive but not yet on ART, over a six-month period. This allowed us to track the trends in miRNA expression in correlation with CD4 count and assess their potential as biomarkers for disease progression and treatment efficacy. In the clinical management of HIV, CD4 cell counts are a cornerstone for assessing immune system status and tracking disease progression. While flow cytometry remains the gold standard for measuring CD4 counts, it requires the analysis to be performed on fresh blood samples, limiting flexibility in sample processing and storage. In contrast, biomarkers such as miRNAs assessed through RT-PCR can be extracted and analyzed at a later time, providing greater practicality and convenience in resource-limited settings. Furthermore, flow cytometry’s reliance on costly equipment and highly trained personnel further restricts its accessibility. Therefore, there is a need for more accessible and cost-effective alternative markers that can be used in monitoring HIV prognosis. Recent studies have shown that serum miRNAs can be used as potential biomarkers in the diagnosis and prognosis of various diseases.

As of November 2024, the total reported number of HIV infections in Türkiye is 48273, with 1567 new diagnoses reported in 2024 (41). Although the HIV prevalence in Türkiye remains low (0.1–0.3%), the number of reported new diagnoses has rapidly increased over the last five years, particularly among the young population, where high infection rates have been observed (42). The number of new HIV diagnoses has tripled in the past decade, and the number of new diagnoses reported in the last five years accounts for 63% of the total number reported to date (43). If this trend continues, the prevalence of HIV in Türkiye could significantly increase and place excessive strain on the Turkish health system.

We found that the expression of hsa-miR-27a-3p was upregulated in early stage of ART (month 1) in HIV patients compared with HIV treatment naïve group; notably, this increase became even more pronounced at later time points (months 2 and 6) when compared to both treatment-naïve patients and healthy individuals. We speculate that in healthy controls, miR-27a levels remain low as the immune system operates in a balanced state without the stress of viral infections. miR-27a plays a role in regulating pathways like EGFR/MEK/ERK, which are involved in cellular proliferation, survival, and differentiation (44). In the absence of viral stress, there is no need for miR-27a upregulation, as these pathways are not over-activated. The basal expression of miR-27a in healthy controls ensures normal cellular function without excessive suppression of these essential processes. However, in HIV-positive patients, the immune system undergoes significant stress, and the virus hijacks host signaling pathways such as EGFR/MEK/ERK to enhance its replication and survival. Ligands like epidermal growth factor (EGF) binding to EGFR initiates auto-phosphorylation of receptor tyrosine kinases, triggering EGFR/MEK/ERK pathways (45). Several viruses, including rhinovirus, influenza, Epstein-Barr virus, cytomegalovirus, and hepatitis C virus activate EGFR to enhance their survival and replication in host cells (46–49). Similarly, HIV may exploit these pathways during its infection cycle.

Interestingly, during HCV infection, MEK/ERK pathway activation can attenuate the IFN-JAK-STAT pathway (50). Reduced miR-27a expression at baseline could therefore facilitate EGFR-mediated attenuation of interferon signaling, aiding HIV persistence. When ART is initiated, viral replication decreases, and the immune system begins to recover. This leads to a gradual upregulation of miR-27a, as observed at months 2 and 6 post-ART which is consistent with previous findings (51). Similarly, Consuegra et al. (45) also demonstrated a significant increase in miR-27a levels in HIV patients compared to the healthy controls. The dynamic regulation of miR-27a in HIV patients reflects the transition from immune dysregulation to recovery under ART. In contrast, the low levels of miR-27a in healthy controls reflect a stable immune environment where its intervention is not required. This difference underscores the adaptive role of miR-27a in regulating host pathways during viral infections and its potential as a biomarker for monitoring immune recovery in HIV-positive patients. Many studies have emphasized miRNAs as critical modulators of the complex interactions between hosts and pathogens. Data from *in vitro* study by Zhang et al. (44) reported the significantly decreased expression of miR-27a in cells infected with Enterovirus 71. Interestingly, overexpression of miR-27a can inhibit Enterovirus 71 replication, as determined by viral titration, qPCR, and Western blot.

Furthermore, upregulation of hsa-miR-548c-3p post-ART 6 months was observed, when compared with healthy individuals and HIV treatment naïve group. Limited data is present about the role of this microRNA in HIV. According to Roth et al. (52) study, miR-548 was identified among the miRNAs selectively upregulated in exosomes derived from HIV-1-infected macrophages compared to uninfected controls, suggesting a potential role in intercellular communication or modulation of host responses during early HIV-1 infection. Similarly, the upregulation of miR-548, identified as a key regulatory miRNA interacting with PLA2G16 in HIV-associated neurocognitive disorder (HAND) study (53). Notably, miR-548d-3p is upregulated in human visceral leishmaniasis, where it suppresses parasite growth within macrophages, highlighting its role in innate immune defense mechanisms (54).

The study conducted by Lin et al. (55) in chronic hepatitis B patients provided valuable insights into the molecular mechanisms underlying inflammatory responses and immune regulation. They found that low levels of miR-548c lead to increased TRIM22 expression, provided valuable insight into the potential molecular mechanisms underlying inflammatory responses and immune regulation. TRIM22 overexpression, in turn, was shown to significantly increase the levels of pro-inflammatory cytokines like IL-1β and IL-8, which are involved in the immune response and inflammation. This supports the idea that miR-548c-3p plays a crucial role in modulating these inflammatory pathways by regulating TRIM22 expression (55). In a study on breast cancer, the expression of Enoyl-CoA hydratase (ECHS1) was reported to be higher in breast cancer cells and tissues. The molecule miR-548-3p was shown to target and control the expression of ECHS1. When miR-548-3p was increased, it slowed down the growth of breast cancer cells by reducing ECHS1 expression (56). In a study on HIV patients, the drug Dolutegravir (an integrase inhibitor) was found to reduce lipid metabolism and increase the expression of Enoyl-CoA hydratase; an enzyme involved in breaking down fats for energy. This change in lipid metabolism could negatively affect the macrophages. Furthermore, HIV infection, combined with the use of Dolutegravir, seems to disrupt healthy macrophage functions in the adipose tissue (fat storage areas) of people living with HIV (PWH). This disruption can lead to metabolic diseases like obesity or diabetes, especially in people who are already at risk. It could also worsen inflammation and other negative effects on the body’s fat storage, making it harder to maintain proper fat tissue balance and immune function (57).

In recent years, miR-124 has emerged as a critical modulator of immunity and inflammation (58). Our findings, which show elevated miR-124 expression at 1 month followed by gradual normalization at 2 and 6 months, are consistent with studies on miR-124’s role in immune regulation during inflammatory conditions, including HIV infection (59, 60). However, several miRNAs, including miR-124, were found to modulate viral spread in T-lymphocytes and HeLa-CCR5 cell lines. Following infection, let-7c, miR-34a, or miR-124 was upregulated, and they targeted and downregulated p21 and TASK1, which eventually led to increased virion release and higher copy number of viral genome transcripts in infected cells, suggesting that HIV-1 could utilize the host miRNA cellular systems to produce a more efficient infection process via blocking the mechanism of innate inhibition (59).

The innate immune system coordinates host defenses through pattern recognition receptors, such as toll-like receptors (TLRs), and miR-124 has been shown to regulate innate immune responses, such as TLR4 activation, as well as the cholinergic anti-inflammatory pathway (61). miR-124 further modulates TLR4-induced cytokine production by targeting signal transducer and activator of transcription 3 (STAT3), reducing IL-6 production, and targeting TNF-α converting enzyme (TACE) to diminish TNF-α release (61). In another study, IL-7 was shown to elevate miR-124 levels and subsequently increase CD95 expression in CD4^+^ T cells from HIV-1-infected individuals, priming these cells for CD95-mediated signals and aiding the maintenance of the HIV-1 reservoir (60). Studies on opioid use, such as morphine’s effect on microglia, and the impact of HIV Tat protein on miR-124 regulation suggested that sustained inflammation and immune activation may contribute to the retention of miR-124 expression even after initial treatment (62). miR-124 serves as a key modulator of both innate and adaptive immune responses, influencing TLR signaling, cytokine production, and macrophage activation. The slight persistence of elevated miR-124 in HIV patients, even after treatment, suggests ongoing immune dysregulation. Long-term data could provide further insights into miR-124 as a biomarker for immune activation in HIV patients and its potential role in predicting treatment responses or complications. The ability of miRNA analyses to distinguish between individuals with the presence or absence of HIV-1 infection has been demonstrated in studies, worldwide (19).

We also investigated the relationship between CD4 count and the expression of four miRNAs: miRNA-124-3p, miRNA-548c-3p, miRNA-3163, and miRNA-27a-3p. The results revealed that miRNA-124-3p had a moderate-to-strong negative correlation with CD4 count, suggesting that higher miRNA-124-3p levels are associated with lower CD4 counts. In contrast, miRNA-548c-3p and miRNA-3163 showed moderate-to-strong positive correlations with CD4 count, indicating a potential role in supporting immune function. No significant correlation was found between miRNA-27a-3p expression and CD4 count. Additionally, weak correlations were observed between the miRNAs and the CD4/CD45 ratio, with miRNA-124-3p showing a negative correlation, while others exhibited weak positive correlations. One-way ANCOVA analysis indicated that miRNA-3163 and miRNA-124-3p significantly influenced CD4 count, highlighting their potential as biomarkers for monitoring immune response during HIV treatment. These findings suggest that miRNA expression could be valuable in predicting treatment outcomes and immune recovery in HIV patients. Despite some efforts to elucidate the relationship, the association of CD8^+^ T cell counts with HIV-related miRNAs, cellular pathways, diseases, and receptors has not been fully clarified. Previous studies have shown a positive correlation between CD8^+^ T cell counts and viral reservoirs (63, 64). Collins et al. (65) opened discussions on the increasing evidence obtained from individuals who spontaneously control infection without antiretroviral therapy, providing a clear rationale for development of a CD8^+^ T cell-based HIV vaccine alongside B cell vaccine studies. Intracellular factors regulating T cell differentiation include miRNAs. miR-16, miR-142–3p, miR-150, miR-142–5p, and miR-15b are the most highly expressed miRNAs in naive CD8^+^ T cells, and all of these miRNAs are seen to undergo downregulation in *in vitro* experiments.

Huang et al. (66) discovered that miR-28, miR-125b, miR-150, miR-223, and miR-382-5p target the 3’UTR region of HIV-1 mRNA in cells, reducing CD4^+^ T cell activation during the resting phase. Monocytes are rich in these anti-HIV-1 miRNAs, which are downregulated in macrophages after differentiation, making macrophages susceptible to HIV-1 infection. The suppression of these miRNAs increases the sensitivity of mononuclear cells to HIV-1 infection. The 3’UTR region of the HIV-1 RNA genome has been identified as a target for miR-196b and miR-1290. Inhibition of these two miRNAs leads to the activation of latent HIV-1, resulting in virus-induced cytolysis and the clearance of latent viral reservoirs with host antiviral immune responses in the presence of antiretroviral therapy.

It is known that HIV-1 infects CD4^+^ T cells, allowing biological processes to occur between them, particularly Th17 cells, in viral replication. The infection of Th17 cells is critical for AIDS pathogenesis and for the virus that remains latent in the body (67). Wiche Salinas et al. (68) found that the hormone receptor (RORC2) could be targeted as a cell-specific target to reduce the loss of Th17 cells resulting from HIV-1 infection.

When examining the treatment process, the role of miRNAs in modulating HIV infection becomes increasingly evident. Specifically, understanding how miRNAs such as miR-27a, miR-3136, miR-548c, and miR-124 respond to acute treatment is crucial for refining and optimizing treatment strategies. These miRNAs have been implicated in various stages of the HIV infection cycle, influencing viral replication, immune response, and inflammation. Their expression profiles during treatment can provide valuable insights into the underlying mechanisms of immune recovery and immune dysregulation. By monitoring the changes in miRNA expression, clinicians could potentially identify biomarkers that predict treatment efficacy, resistance, and long-term immune recovery, ultimately aiding in the development of more personalized and effective therapeutic approaches for HIV patients.

## 5 Conclusion

This study underscores the potential of plasma miRNAs as reliable biomarkers for HIV prognosis, with hsa-miR-3163 emerging as the most promising candidate. Its significant prognostic accuracy and strong association with CD4 count highlight its utility in monitoring disease progression and optimizing therapeutic interventions. These findings pave the way for further research aimed at validating these results in larger cohorts and elucidating the mechanistic roles of these miRNAs in HIV pathogenesis and immune regulation.

Integrating miRNA analysis with clinical data could advance personalized antiretroviral therapy, enabling tailored treatment approaches. Targeting these miRNAs through activation or inhibition may help restore CD4^+^ cell populations to optimal levels, offering a novel strategy to enhance immune function. Moreover, these miRNAs hold promise as prognostic biomarkers for monitoring HIV-1 infection progression and evaluating treatment efficacy. Future research should focus on establishing robust quantitative correlations and exploring the therapeutic potential of modulating miRNA activity. Such efforts could significantly improve the diagnosis, early management, and long-term outcomes of HIV.

## Data Availability

The original contributions presented in this study are included in the article. Due to the nature of the clinical data and ethical considerations, additional supporting data are available from the corresponding author upon reasonable request and with appropriate institutional approval.
